# Eicosapentaenoic and Docosahexaenoic Acid Supplementation Increases HDL Content in n-3 Fatty Acids and Improves Endothelial Function in Hypertriglyceridemic Patients

**DOI:** 10.3390/ijms24065390

**Published:** 2023-03-11

**Authors:** Paola Peña-de-la-Sancha, Adolfo Muñoz-García, Nilda Espínola-Zavaleta, Rocío Bautista-Pérez, Ana María Mejía, María Luna-Luna, Victoria López-Olmos, José-Manuel Rodríguez-Pérez, José-Manuel Fragoso, Elizabeth Carreón-Torres, Óscar Pérez-Méndez

**Affiliations:** 1 Department of Molecular Biology, Instituto Nacional de Cardiología Ignacio Chávez, Mexico City 14080, Mexico; 2 Department of Nuclear Medicine, Instituto Nacional de Cardiología Ignacio Chávez, Mexico City 14080, Mexico; 3 Department of Echocardiography, ABC Medical Center, I.A.P, Mexico City 01120, Mexico; 4 Blood Bank, Instituto Nacional de Cardiología Ignacio Chávez, Mexico City 14080, Mexico; 5 Tecnologico de Monterrey, School of Engineering and Sciences, Mexico City 14380, Mexico

**Keywords:** omega-3 fatty acids, HDL, hypertrygliceridemia, endothelial function, HDL vascular properties

## Abstract

High-density lipoproteins (HDLs) are known to enhance vascular function through different mechanisms, including the delivery of functional lipids to endothelial cells. Therefore, we hypothesized that omega-3 (n-3) eicosapentaenoic acid (EPA) and docosahexaenoic acid (DHA) content of HDLs would improve the beneficial vascular effects of these lipoproteins. To explore this hypothesis, we performed a placebo-controlled crossover clinical trial in 18 hypertriglyceridemic patients without clinical symptoms of coronary heart disease who received highly purified EPA 460 mg and DHA 380 mg, twice a day for 5 weeks or placebo. After 5 weeks of treatment, patients followed a 4-week washout period before crossover. HDLs were isolated using sequential ultracentrifugation for characterization and determination of fatty acid content. Our results showed that n-3 supplementation induced a significant decrease in body mass index, waist circumference as well as triglycerides and HDL-triglyceride plasma concentrations, whilst HDL-cholesterol and HDL-phospholipids significantly increased. On the other hand, HDL, EPA, and DHA content increased by 131% and 62%, respectively, whereas 3 omega-6 fatty acids significantly decreased in HDL structures. In addition, the EPA-to-arachidonic acid (AA) ratio increased more than twice within HDLs suggesting an improvement in their anti-inflammatory properties. All HDL-fatty acid modifications did not affect the size distribution or the stability of these lipoproteins and were concomitant with a significant increase in endothelial function assessed using a flow-mediated dilatation test (FMD) after n-3 supplementation. However, endothelial function was not improved in vitro using a model of rat aortic rings co-incubated with HDLs before or after treatment with n-3. These results suggest a beneficial effect of n-3 on endothelial function through a mechanism independent of HDL composition. In conclusion, we demonstrated that EPA and DHA supplementation for 5 weeks improved vascular function in hypertriglyceridemic patients, and induced enrichment of HDLs with EPA and DHA to the detriment of some n-6 fatty acids. The significant increase in the EPA-to-AA ratio in HDLs is indicative of a more anti-inflammatory profile of these lipoproteins.

## 1. Introduction

Long-term prospective cohort studies have consistently demonstrated an association between higher intakes of omega-3 (n-3) fatty acids, particularly eicosapentaenoic acid (EPA, 20:5 n-3) and docosahexaenoic acid (DHA, 22:6 n-3), and a lower risk of developing coronary artery disease (CAD) [1,2,3,4,5,6]. EPA and DHA modulate some of the most important known risk factors of CAD, such as blood lipids, blood pressure, heart rate, heart rate variability, platelet aggregation, inflammation, and endothelial function [6]. Of particular interest, endothelial dysfunction is one of the earliest events in the pathological development of atherosclerotic diseases [6,7]. Accordingly, prospective studies have shown that flow-mediated dilation (FMD), the gold standard for measuring endothelial dysfunction in vivo, is an independent predictor of cardiovascular events, such as heart attack or stroke [6,7,8,9,10,11]. Therefore, therapies targeted to improve endothelial dysfunction, such as EPA and DHA supplementation, may report important benefits to subjects at risk of CAD.

In this context, high-density lipoproteins (HDLs) have been described as lipoproteins with beneficial effects on endothelial function [12,13,14], probably associated with their EPA and DHA content [15]. HDLs are heterogeneous complexes of proteins and lipids that have been proposed to protect against cardiovascular disease through different mechanisms, including the reverse transport of cholesterol (RTC) [16]. It has also been described that HDLs induce endothelial nitric oxide synthase (eNOS) stability and phosphorylation, increasing its halftime and abundance [17,18]. Besides cholesterol efflux, we have recently demonstrated that HDLs also promote the influx of sphingomyelin and cholesterol, which improves endothelial cells in vitro [18]. Such observations suggest that HDLs are vectors that drive lipids to peripheral cells for structuring their membranes and other cellular functions [16,18]. Following this idea, we postulate that HDLs may carry n-3 fatty acids to endothelial cells in vivo, thus enhancing the functionality of this tissue. We, therefore, isolated HDLs from patients treated with EPA and DHA and determined whether these lipoproteins effectively become enriched in n-3 fatty acids during treatment. We further explored whether EPA and DHA induce an improvement of endothelial function in vivo and the potential contribution of HDLs to this beneficial effect.

## 2. Results

### 2.1. Patients Included in the Study

**Figure 1:**
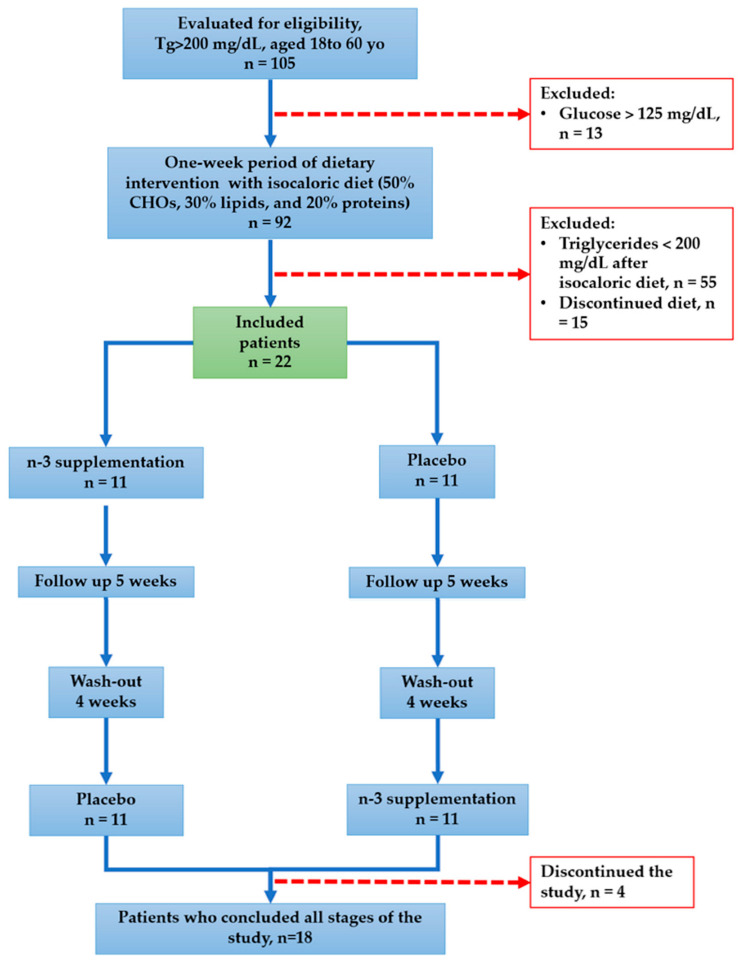
Flow chart of the study.

We evaluated for eligibility 105 individuals who were initially candidates for the study. Eighty-three subjects were excluded; 13 because of glucose above 125 mg/dL, 55 subjects reduced their triglycerides below 200 mg/dL, and 15 individuals abandoned the study during the dietary intervention. Then, 22 hypertriglyceridemic patients were enrolled. During the following stages of the trial, 4 patients withdrew from the study. Consequently, we reported the results of 18 patients who concluded all the phases of the protocol (Figure 1).

### 2.2. Anthropometric and Biochemical Characteristics during EPA and DHA Supplementation

Anthropometric and biochemical data of patients before and after n-3 fatty acids or placebo are presented in Table 1. Slight decreases in body mass index (BMI) were observed in either, n-3 fatty acids, or placebo, but only the former reached statistical significance. Concomitantly, body fat mass diminished during treatment with EPA and DHA. Importantly, energy intake did not change during placebo or n-3 supplementation (Supplementary Table S1). Other anthropometric measurements remained unchanged after interventions. As expected, the median of triglycerides decreased, whereas HDL-cholesterol increased with n-3 supplementation (Table 1).

### 2.3. HDL Characterization

#### 2.3.1. HDL Subclasses and Lipid Content

In addition to the increase in HDL-cholesterol plasma concentrations after n-3 supplementation, HDL-phospholipids were significantly augmented by 27%, whereas HDL-triglycerides diminished by about 35% (Table 1). These results suggest that n-3 supplementation is associated with structural modifications of HDL particles. Therefore, we determined the relative proportion of HDL subclasses and their lipid content. The relative proportion of HDL protein remained unchanged along the subclasses with either placebo or n-3 supplementation (Table 2).

Cholesterol tended to increase in all HDL subclasses, but reached statistical significance only for small particles HDL3b and HDL3c, indicating that n-3 supplementation did not affect size distribution and had a mild effect on lipid content in HDLs (Supplementary Table S2). In contrast, the quality of fatty acids was modified; as expected, HDL content of EPA and DHA increased 131.0% and 61.6%, respectively, after 5 weeks of n-3 supplementation.

Besides the increase in EPA and DHA content in HDLs, three n-6 fatty acids significantly decreased, particularly cis-5,8,11-eicosatrienoic acid (20:3 n-6) precursor of arachidonic acid (AA, 20:4 n-6), and two fatty acids derived from AA, cis-7,10,13,16-docosatetraenoic acid (22:4 n-6), and cis-4,7,10,13,16-docosapentaenoic acid (22:5 n-6) (Table 3). Consequently, the EPA-to-AA ratio in HDLs increased more than twice the basal level after n-3 supplementation (from 0.0503 ± 0.033 to 0.128 ± 0.091, *p* = 0.001). Moreover, the DHA-to-AA ratio in HDLs drastically increased, from 0.074 ± 0.017 in basal conditions to 0.400 ± 0.153 after n-3 supplementation.

#### 2.3.2. HDL Stability

Polyunsaturated fatty acids are more fluid than mono or saturated fatty acids and consequently, the increase in n-3 content in HDLs may compromise the stability of the particle. Therefore, we analyzed HDL stability by estimating the proportion of apolipoproteins that abandoned the lipid environment of lipoprotein at 8 h in the presence of urea (percentage of denaturation), as previously described [19]. The median of the percentage of HDL denaturation in basal conditions was 69.5% [33.2–90.2%] and remained similar after the n-3 supplementation (75.7% [38.8–91.4%], *p* > 0.05), indicating that the stability of HDLs was not affected by the increased proportion of n-3 fatty acids described above.

### 2.4. Effect n-3 Supplementation on Vascular Function

#### 2.4.1. Flow-Mediated Vasodilation In Vivo

We determined the endothelial function in vivo by flow-mediated vasodilation (FMD); results are shown in Table 4. The internal diameter of the brachial artery after 1 min of hyperemia increased significantly in hypertriglyceridemic patients during supplementation with n-3. In contrast, FMD remained similar before and after the placebo (Table 4).

#### 2.4.2. Endothelial-Mediated Vasodilation In Vitro

**Figure 2:**
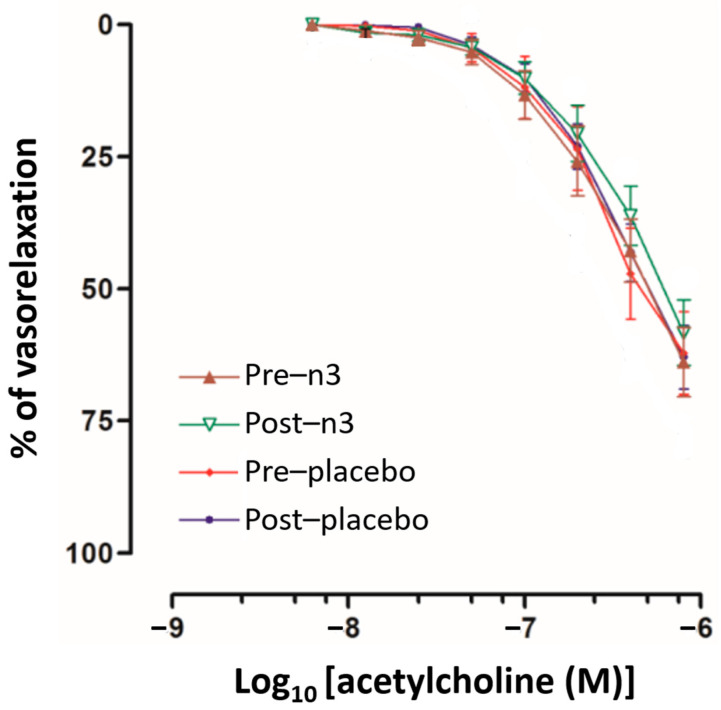
Endothelial-dependent vasorelaxation in the function of increasing doses of acetylcholine of precontracted rat aorta rings. Percentages were determined using the baseline of the contraction of vascular smooth muscle with epinephrine 3 × 10^−4^ M. HDL isolated from the plasma of the 18 patients at each of the 4 stages of the study were tested in duplicate. n-3: EPA and DHA supplementation.

To explore whether HDLs contribute to FMD enhancement observed in vivo, we determined the endothelial-dependent vasorelaxation using a model of rat aorta rings incubated with HDL isolated from patients’ plasma; these results are shown in Figure 2. As observed, the vasorelaxation induced by increasing doses of acetylcholine is similar in the presence of HDLs obtained after n-3 supplementation or placebo, before or after intervention.

## 3. Discussion

In this study, we explored whether the beneficial effects of EPA and DHA supplementation on vascular function might be linked to HDL structure. Both HDLs and-3 fatty acids have been shown to improve endothelial function [6,17,18,20], and such a function may be interrelated. In this context, previous reports have demonstrated the capacity of HDLs to induce Ser1177 phosphorylation of eNOS (indispensable to enzyme activation) related to the lipid content of these lipoproteins [18]. In addition, the capacity of HDLs to regulate ICAM-1 expression in cultured endothelial cells is also dependent on their lipid content [18]. These observations strongly suggest that phospholipids and triglycerides structured with EPA or DHA may be driven by HDLs into endothelial cells, thus facilitating the beneficial effects on the vascular function of these polyunsaturated fatty acids.

Our results showed that 2 g/d of EPA and DHA effectively improved endothelial function assessed using FMD. This beneficial effect of n-3 on vascular function is still controversial [20,21,22]. Treatment with omega 3 failed to improve FMD in a group of 38 patients with T2DM, HbA1c < 7%, high cardiovascular risk, and optimal medical therapy for hypertension and plasma lipids [21]. In that study, patients were about 20 years older than individuals included in our trial. Moreover, we focused on individuals who did not take any medication and with glucose levels below 125 mg/dL. Consequently, our data support the idea that n3 supplementation is useful for improving FMD in patients with a vascular function relatively preserved, as observed in this and other studies [20,23]. Conversely, when advanced atherosclerotic disease is already established, EPA and DHA do not seem to report a benefit on vascular function [21]. In this context, n-3 supplementation is likely useful to prevent endothelial dysfunction rather than to treat wounded vessels. Long-term prospective studies are needed to address this hypothesis.

Once we demonstrated the beneficial effect of EPA and DHA on vascular function, the next step was to explore whether HDLs effectively carried both n-3 fatty acids. Our results showed a significant increase in EPA and DHA as components of lipids carried by HDLs. Such increments were similar to that observed in the plasma of patients with high cardiovascular risk treated with 1800 mg/d of n-3 as fish oil, and who reached 220.3% and 68.3% of EPA and DHA increase in plasma, respectively [15]; this study also demonstrated a modification of HDL subclasses induced by n-3 supplementation. Conversely, we did not observe any change in HDL size distribution or in lipids of HDL subclasses, probably because our study group was more homogeneous in terms of risk factors than the previous report [15].

Besides EPA and DHA, three n-6 fatty acids significantly decreased during n-3 supplementation, the precursor of arachidonic acid, and two of its metabolites as mentioned in the results section. This observation suggests that HDLs, EPA, and DHA compete with other long-chain fatty acids for key enzymes related to inflammatory processes. Of particular interest, is the increase in the EPA-to-AA ratio of about twice the basal value. EPA and AA may compete for the active site of cyclooxygenases (COX’s) since both fatty acids are substrates of these enzymes [24]. However, EPA-derived prostaglandins (PG) are minimally inflammatory or may even have anti-inflammatory properties, such as PGE3 which may selectively promote M2a polarization, while inhibiting M1 [25]. Therefore, the increased proportion of the EPA-to-AA ratio is likely to enhance the anti-inflammatory properties of HDLs during n-3 supplementation. This evidence is consistent with the early use of n-3 fatty acids to lower interleukin-1 and tumor necrosis factor [26] and also agrees with the opposite statistical association of EPA and AA with the risk of CAD in young Chinese patients [27]; AA was associated with an increased risk of CAD, whereas EPA seemed to play a protective role against the disease. Consequently, the ratio of EPA-to-AA can increase the predictive value for diagnosing CAD more than EPA or AA alone [27]. Based on this evidence, the huge increase in EPA proportion with respect to AA in HDLs further supports the idea that n-3 supplementation enhances the atheroprotective properties of these lipoproteins. Moreover, DHA may contribute to the increase the anti-inflammatory properties of HDLs since this fatty acid, as well as EPA, are precursors of resolvins (inflammation-resolving mediators) via the lipoxygenase pathway [24]. Finally, it cannot be discarded that the reduction in some other n-6 fatty acids in HDL may also contribute to the beneficial effects of n-3 supplementation [15]. This issue needs to be addressed in further studies.

Polyunsaturated fatty acids are more fluid than monounsaturated or saturated fatty acids; consequently, the increase in n-3 content in HDLs may compromise the stability of the particle via the modification of its surface tension, which is fundamental for the metabolism and function of these lipoproteins [28,29]. This relevant issue had to be considered since the stability of HDLs could limit their potential beneficial effects on vascular function. Then, to establish whether the increased proportion of n-3 in HDLs may affect the stability of the particle, we analyzed their capacity of apolipoprotein to remain associated with HDL-lipids in the presence of urea. Our results clearly showed that the increased proportions of n-3 fatty acids after supplementation did not affect the stability of HDLs. Of notice, EPA and DHA increased, but concomitantly, other polyunsaturated fatty acids, such as 20:3 n-6, 22:4 n-6 and 22:5 n-6, significantly decreased, compensating somehow for the fluidity of the particle. This speculative explanation needs to be explored in future studies.

Once we confirmed that HDLs were enriched with n-3 fatty acids, we further explored the possibility that EPA and DHA contribute to endothelial function improvement via these lipoproteins. For this, we pre-incubated rat aorta rings with HDLs from patients and then contracted them with epinephrine. The addition of increasing doses of acetylcholine induces the endothelium-dependent relaxation of vascular smooth muscle [30]. Using this model, we were not able to demonstrate that HDL enriched with EPA and DHA enhanced endothelial function. It should be considered that vascular tissue was incubated with HDL for only 1 h; even if the exchange of HDL-lipids with cells occurs within the first 60 min [18], the intracellular signaling of n-3 [6] is likely to be a process of longer duration. Therefore, we cannot still discard the possibility that the beneficial effects of HDLs on endothelial function are linked to their EPA and DHA content, but in a long-term manner. Other models of endothelial function suitable for longer incubation times should be developed to address this issue.

Finally, long-term effects of EPA and DHA that have been observed in some tissues [31] may have persisted through the wash-out period in patients who initiated in the n-3 supplementation arm. Moreover, an isocaloric diet may also provide some beneficial effects during the study. Consequently, some parameters, such as HDL-triglycerides and FMD, tended to be different before the placebo with respect to the pre-n-3 supplementation values, and the positive effects tended to increase by the end of the crossover period. Even if such differences did not reach statistical significance, they are inherent to the design of the trial and should be recognized as a weakness of this study.

## 4. Materials and Methods

### 4.1. Patients and Study Design

One hundred and five non-smoking consecutive male volunteers from the blood bank of the Instituto Nacional de Cardiologia “Ignacio Chavez” were screened for this study. Subjects with triglycerides plasma levels > 200 mg/dL, who were not taking any anti-dyslipidemic drug, and did not have dysthyroidism, liver disease, autoimmune or congenital heart disease as determined using biochemical analyses or medical history, were invited to the next step of the study. Volunteers completed a survey regarding lifestyle factors, medication, and diet habits, which included a 24 h questionnaire. Then, subjects were instructed to follow an isocaloric diet personally designed to reach a balanced intake of 50, 30, and 20% of total calories from carbohydrates, lipids, and proteins, respectively. Subjects whose fasting triglyceride plasma levels decreased below 200 mg/dL after one week of an isocaloric diet were excluded. Eighteen subjects were finally included to complete the trial. The Isocaloric diet was continued during the study (Figure 1).

We performed a single-blind randomized crossover study that was placebo-controlled. Hypertriglyceridemic patients received EPA 460 mg and DHA 380 mg twice a day for 5 weeks or placebo, followed by a washout period of 4 weeks before crossover. Anthropometric measures, blood pressure, flow-mediated vasodilation, biochemical profile and HDL characterization were performed before and after placebo or EPA + DHA supplementation.

The study was performed in accordance with the appropriate version of the Declaration of Helsinki and approved by the Ethics Committee from the Instituto Nacional de Cardiología “Ignacio Chávez” with registration numbers 17-996 and 22-1338. All the patients gave their written informed consent prior to the recruitment.

### 4.2. Laboratory Analysis

After 12 h of overnight fasting, blood samples were drawn in EDTA or dry tubes, and centrifuged for 15 min at 1300× *g* within 15 min after collection. Plasma and serum were separated into 500 µL aliquots and then either immediately analyzed or frozen at −80 °C until analysis. Glucose, cholesterol, and triglycerides plasma concentrations were determined by commercially available enzymatic/colorimetric assays (Randox Labs. Ltd., Crumlin, Co., Antrim, Northern Ireland, UK). The phosphotungstic acid-Mg + 2 method was used to precipitate apo B-containing lipoproteins before quantifying HDL-cholesterol (HDL-C) and HDL-triglycerides (HDL-Tg). HDL-phospholipids (HDL-Pho) concentrations were quantified using a phospholipase C method (Wako Chemicals, Richmond, VA, USA).

### 4.3. HDL Subclasses Composition Assessment

HDLs were isolated using ultracentrifugation and analyzed as previously described [32]. Briefly, after recovery, HDLs were further separated by their hydrodynamic diameter; 25 µg of HDL protein were run in a non-denaturing 3–30% gradient polyacrylamide gel electrophoresis. Gels were enzymatically stained for cholesterol or triglycerides and scanned to obtain the densitograms for these HDL components. Thereafter, gels were re-stained with Coomassie blue R-250 to detect proteins. HDL subclasses and size intervals were established using globular proteins as diameter references (high-molecular-weight calibration kit, Amersham Pharmacia Biotech, Buckinghamshire, UK). Each subclass was measured using VisionWorks 8.20 version and expressed as the percentage of total HDL-protein or lipid area of the densitogram (7.94 to 13.59 nm) [32,33]

Total fatty acids in HDL were determined using gas chromatography after the hydrolysis of lipids [34] using an external service provider (OmegaQuant HQ, Sioux Falls, SD). For this, 50 μg of HDL protein was placed and dried in a paper support containing an antioxidant preservative following the service provider’s instructions. Data were reported as the percentage of the total fatty acids.

### 4.4. HDL Stability

The stability of HDLs was determined using the Trp fluorescence red-shift during the denaturation of isolated lipoproteins at a final concentration of 30 μg/mL, in PBS-8M urea, pH 7.4 as previously described [19]. Briefly, Trp intrinsic fluorescence emission was measured at 330, 344 and 365 nm (Emλ30, Emλ344, and Emλ365, respectively) using an LS55 Perkin-Elmer fluorescence spectrophotometer (Walthman, MA, USA) at room temperature, with an excitation wavelength (λ_ex_) of 280 nm and bandwidth of 5.5 nm. Then, the ratio of fluorescence intensities ($r_{fi}$) was calculated as:

$$r_{fi}=\frac{\left(Em\lambda 344+Em\lambda 365\right)}{Em\lambda 330}$$

For every sample of isolated HDL, $r_{fi}$ was determined at the initial time (t = 0 h), at 8 h, and finally at 26 h. The $r_{fi}$ at 0 h was considered as 0% of denaturation, whereas the $r_{fi}$ value at 26 h was set as 100% of the measurable denaturation of HDLs [19]. The proportional percentage of denaturation at 8 h represents the stability of the particle, i.e., the lower the percentage at 8 h, the higher the stability of the particle.

### 4.5. Flow-Mediated Vasodilation

Endothelial function determined using flow-mediated vasodilation (FMD) was determined using the ultrasound method as previously described [35,36]. The brachial artery diameter was measured in the longitudinal plane, 5 cm above the antecubital fossa after at least 30 min of resting position. The site of measurement was marked on the skin, and the arm was held in the same position during the entire study. Reactive hyperemia was induced by inflating a blood pressure cuff to 200 mmHg for 5 min. The internal diameter of the brachial artery was measured 3 times and averages were taken at rest and in the first minute after deflating the cuff. FMD was defined as the change in the internal diameter of the brachial artery during reactive hyperemia compared to the basal diameter and expressed as a percentage [35,36].

### 4.6. Vascular Reactivity of Aorta Rings

The potential effect of HDLs on endothelial function was explored using aorta rings from Wistar rats in an organ bath, as previously described [30,33]. Aorta rings were incubated with isolated HDL to a final concentration of 50 mg/dL of cholesterol in Krebs solution and 95% O_2_/5% CO_2_ continuous flow at 37 °C, 1 h previous to the test. After an equilibrium period, aorta rings were pre-contracted with phenylephrine 3 × 10^−4^ M. The endothelium-mediated relaxation (vasodilation) was evaluated by the increasing addition of acetylcholine from 5 × 10^−9^ to 8 × 10^−7^ M of final concentration. Vascular contractions were measured by means of an FT–03 Grass Force Displacement Transducer and recorded on a Grass Polygraph (Model 7D, Grass Medical Instruments, Quincy, MA, USA). All experiments were run in duplicate, and results were expressed as the percentage of relaxation with respect to pre-contraction with phenylephrine [30,33].

## 5. Conclusions

In this study, we demonstrated that EPA 460 mg and DHA 380 mg twice a day for 5 weeks improved vascular function assessed using flow-mediated dilation in hypertriglyceridemic patients without clinical symptoms of coronary heart disease and who were not receiving other drugs. Vascular function improvement was concomitant with an enrichment of HDL with EPA and DHA that did not affect the stability of these lipoproteins. The significant increase in the EPA-to-AA ratio in HDLs may contribute to the enhancement of the anti-inflammatory effects of these lipoproteins.

## Figures and Tables

**Table 1:** Anthropometric and biochemical characteristics of included patients, before and after supplementation with EPA and DHA or placebo.

	Pre-n-3	Post-n-3	Pre-Placebo	Post-Placebo
Age (years)	42.5 ± 6.2			
SBP (mmHg)	117.0 ± 8.2	115.8 ± 7.9	119.8 ± 8.0	115.9 ± 10.5
DBP (mmHg)	81.3 ± 4.2	80.4 ± 5.8	81.1 ± 4.0	81.1 ± 3.3
Body fatty mass (%)	30.6 ± 4.9	29.4 ± 4.0 *	30.2 ± 4.7	29.6 ± 4.6
Waist (cm)	102.3 ± 10.2	98.9 ± 9.1	101.1 ± 9.5	99.4 ± 9.1
BMI (kg/m^2^)	30.9 ± 4.2	30.4 ± 3.9 *	30.6 ± 4.2	30.4 ± 4.0
Glucose (mg/dL)	98.2 ± 13.1	97.8 ± 15.0	97.9 ± 12.6	100.0 ± 13.3
Triglycerides (mg/dL)	316.4 [257.3–633.9]	235.3 [176.5–300.4] **	287.5 [206.6–418.4]	261.7 [221.9–395.7]
Cholesterol (mg/dL)	199.0 ± 39.1	215.9 ± 53.5	213.4 ± 67.7	206.8 ± 37.6
Non-HDL-cholesterol (mg/dL)	157.3 ± 42.0	170.2 ± 55.2	171.8 ± 69.0	164.9 ± 37.2
HDL-cholesterol (mg/dL)	41.8 ± 8.2	49.2 ± 10.5 *	41.8 ± 9.3	42.3 ± 9.4
HDL-phospholipids (mg/dL)	56.2 ± 17.9	71.5 ± 21.3 *	55.0 ± 17.8	55.3 ± 20.8
HDL-triglycerides (mg/dL)	11.2 [8.0–13.8]	7.32 [5.3–10.9] **	10.9 [7.3–13.9]	10.5 [8.5–12.9]

Data with normal distribution and non-normal distribution are represented as mean ± SD and median [inter-quartile interval], respectively. n-3: EPA and DHA supplementation. * Student’s *t* test or ** Mann–Whitney U test *p* < 0.05 compared to pre-supplementation condition. SBP: systolic blood pressure. DBP: diastolic blood pressure. BMI: body mass index.

**Table 2:** Relative proportion of HDL subclasses determined by protein content.

HDL Subclass *	Pre-n-3	Post-n-3	Pre-Placebo	Post-Placebo
HDL2b	14.2 ± 9.6	15.5 ± 12.1	13.0 ± 9.9	13.5 ± 10.7
HDL2a	6.9 ± 2.3	7.4 ± 3.0	7.2 ± 3.1	6.9 ± 2.5
HDL3a	30.2 ± 6.2	29.9 ± 6.4	29.4 ± 8.8	31.9 ± 6.4
HDL3b	19.0 ± 5.3	18.9 ± 5.9	20.5 ± 4.6	20.4 ± 6.7
HDL3c	29.5 ± 11.2	28.3 ± 13.4	30.2 ± 12.7	27.2 ± 10.9

* Data are represented as mean ± SD of the percentage of HDL-protein. n-3: EPA and DHA supplementation. * Student’s *t* test *p* < 0.05.

**Table 3:** Percentage of fatty acids contained in HDLs before and after supplementation with EPA and DHA.

	Pre-n-3	Post-n-3	Δ	% Change	*p* *
Saturated					
14:0	0.61 ± 0.17	0.59 ± 0.21	−0.02	−3.3	0.08
16:0	23.55 ± 1.73	23.18 ± 1.03	−0.37	−1.7	0.766
18:0	10.03 ± 1.33	9.70 ± 0.99	−0.33	−3.3	0.337
20:0	0.24 ± 0.05	0.22 ± 0.06	−0.02	−8.3	0.328
22:0	0.88 ± 0.24	0.82 ± 0.23	−0.06	−6.8	0.388
Monounsaturated					
18:1 n-9	18.21 ± 2.34	17.29 ± 2.44	−0.93	−5.1	0.877
20:1 n-9	0.16 ± 0.03	0.15 ± 0.04	−0.01	−6.3	0.157
24:1 n-9	0.24 ± 0.08	0.27 ± 0.09	0.03	12.5	0.358
Polyunsaturated					
n-3					
18:3 n-3	0.52 ± 0.14	0.59 ± 0.19	0.07	13.5	0.211
**20:5 n-3**	0.42 ± 0.28	0.97 ± 0.68	0.55	131.0	0.002
22:5 n-3	0.59 ± 0.11	0.73 ± 0.17	0.14	23.7	0.001
**22:6 n-3**	1.85 ± 0.60	2.99 ± 0.88	1.14	61.6	0.000
n-6					
18:2 n-6	28.16 ± 2.98	28.87 ± 3.14	0.71	2.5	0.493
18:3 n-6	0.33 ± 0.11	0.31 ± 0.12	−0.02	−6.1	0.431
20:2 n-6	0.24 ± 0.06	0.26 ± 0.04	0.02	8.3	0.131
20:3 n-6	2.29 ± 0.48	2.00 ± 0.46	−0.29	−12.7	0.001
20:4 n-6	8.13 ± 1.55	7.82 ± 1.55	−0.31	−3.8	0.309
22:4 n-6	0.37 ± 0.11	0.25 ± 0.06	−0.12	−32.4	0.001
22:5 n-6	0.30 ± 0.08	0.23 ± 0.08	−0.06	−23.3	0.005

Data are represented as mean ± SD of the percentage of fatty acids contained in HDLs. n-3: EPA and DHA supplementation. * Paired Student’s *t* test. Bold letters indicate EPA (20:5 n-3) and DHA (22:6 n-3).

**Table 4:** Flow-mediated vasodilation (FMD) measurements before and after n-3 supplementation or placebo.

	Pre-n-3	Post-n-3	Pre-Placebo	Post-Placebo
Diameter at rest (mm)	4.30 ± 0.39	4.45 ± 0.42	4.23 ± 0.40	4.22 ± 0.51
Diameter at 1 min (mm)	4.72 ± 0.37	4.96 ± 0.47 *	4.60 ± 0.47	4.61 ± 0.48
FMD at 1 min (%)	7.67 ± 2.88	10.80 ± 3.99 **	8.20 ± 4.38	8.58 ± 3.85

Data are represented as mean ± SD. n-3: EPA and DHA supplementation. Paired Student’s *t* test * *p* = 0.005 and ** *p* = 0.021, compared to Pre-n-3 condition.

## Data Availability

The data presented in this study are available on request from the corresponding author. The data are not publicly available due to privacy of personal information.

## Supplementary Materials

The supporting information can be downloaded at: https://www.mdpi.com/article/10.3390/ijms24065390/s1.
