# Supplementary material for: Eicosapentaenoic and Docosahexaenoic Acid Supplementation Increases HDL Content in n-3 Fatty Acids and Improves Endothelial Function in Hypertriglyceridemic Patients
Source: Int J Mol Sci. 2023 Mar 11;24(6):5390. doi: 10.3390/ijms24065390 (PMC10049536; doi:10.3390/ijms24065390)
Supplement: Supplementary file 1 [file ijms-24-05390-s001.zip › ijms-2275163-supplementary.pdf]

Table S1. Patients' energy consumption along the study.

	Pre-n-3	Post-n-3	Pre-placebo	Post-placebo
Energy (kcal/day)	2213.8 ± 516.1	2089.7 ± 362.0	2165.1 ± 422.5	2164.9 ± 475.9
Carbohydrates (g/day)	298.1 ± 100.2	281.8 ± 69.3	272.9 ± 67.9	293.9 ± 59.9
Proteins (g/day)	95.8 ± 27.3	86.9 ± 20.2	103.6 ± 51.8	89.3 ± 35.2
Lipids (g/day)	72.7 ± 23.3	68.7 ± 16.4	73.4 ± 22.0	71.1 ± 25.2

Data are represented as mean ± SD. No significant difference was observed along the stages of the study.

Table S2. Lipid composition of HDL subclasses.

Lipids of HDL-subclass	Pre-n-3	Post-n-3	*p	Pre-placebo	Post-placebo	*p
Cholesterol (mg/dL)						
HDL-2b	13.2 ± 6.1	15.5 ± 8.4	0.222	10.2 ± 6.6	12.5 ± 6.0	0.138
HDL-2a	3.7 ± 1.6	4.0 ± 1.5	0.316	3.7 ± 1.8	3.9 ± 2.1	0.559
HDL-3a	10.7 ± 3.2	12.0 ± 4.0	0.213	11.1 ± 5.1	10.2 ± 4.1	0.266
HDL-3b	6.3 ± 2.2	7.7 ± 2.5	0.031	6.5 ± 2.6	6.4 ± 2.1	0.823
HDL-3c	7.9 ± 4.9	10.3 ± 7.1	0.038	10.0 ± 6.7	9.5 ± 6.4	0.700
Triglycerides (mg/dL)						
HDL-2b	4.2 ± 3.1	3.9 ± 3.0	0.591	2.9 ± 3.5	4.4 ± 5.1	0.081
HDL-2a	1.2 ± 1.1	1.0 ± 0.9	0.525	1.0 ± 0.7	1.2 ± 1.4	0.546
HDL-3a	3.1 ± 2.1	2.7 ± 2.2	0.338	2.8 ± 2.5	2.9 ± 2.9	0.820
HDL-3b	1.5 ± 0.7	1.2 ± 0.7	0.145	2.1 ± 1.9	1.8 ± 1.9	0.073
HDL-3c	3.2 ± 3.0	2.6 ± 2.9	0.050	4.7 ± 9.0	3.2 ± 4.9	0.153

Data are represented as mean ± SD of HDL-lipids plasma concentration. n-3: EPA and DHA supplementation. Student's paired t test.
